# Macular retinal and choroidal thickness in unilateral relentless placoid chorioretinitis analyzed by swept-source optical coherence tomography

**DOI:** 10.1186/s12348-014-0024-x

**Published:** 2014-10-02

**Authors:** Rosa Dolz-Marco, Álvaro Rodríguez-Ratón, Pablo Hernández-Martínez, Isabel Pascual-Camps, María Andreu-Fenoll, Roberto Gallego-Pinazo

**Affiliations:** 1 Unit of Macula, Department of Ophthalmology, La Fe University and Polytechnic Hospital, Av. Salvador Abril Martorell, no. 106, Valencia 46026, Spain; 2 San Eloy Hospital, Bilbao 48902, Spain

**Keywords:** Choroidal thickness, Uveitis, Relentless placoid chorioretinitis, Swept-source OCT

## Abstract

**Background:**

The purpose of this study is to evaluate the retinal and choroidal thickness of the macular region in patients with unilateral relentless placoid chorioretinitis (RPC) and macular involvement. Patients diagnosed with RPC affecting only one eye underwent a comprehensive ophthalmologic examination including best-corrected visual acuity (BCVA), axial length (AL) measurement, slit-lamp examination, and color fundus and autofluorescence photography. The macular region was scanned by swept-source optical coherence tomography in the 1,050-nm wavelength. Automated segmentations of the retina and the choroid were used to obtain the corresponding thickness values.

**Results:**

A total number of three patients (two men and one woman; age range 17 to 62 years) were included. Eyes with clinically evident RPC had a mean AL of 24.62 ± 0.11 mm, whereas in the clinically healthy fellow eyes, the mean AL was 24.65 ± 0.03 (*p* = 0.70). The mean BCVA was 0.93 ± 0.16 in eyes with RPC, and 1.0 in all the fellow eyes (*p* = 0.70). Slit-lamp examination did not reveal any sign of vitreous inflammation in any cases. The mean macular retinal thickness was 288.10 ± 10.22 μm in eyes with RPC, and 300.30 ± 7.17 μm in the healthy fellow eyes (*p* = 0.20). The mean central choroidal thickness was 260.70 ± 140.60 μm in eyes with RPC, and 262.30 ± 123.10 μm in the fellow eyes (*p* = 0.99). The mean macular choroidal thickness was 248.60 ± 128.40 and 255.10 ± 123.60 μm, respectively (*p* = 0.99).

**Conclusions:**

The pathogenesis of RPC remains unknown. No changes in the retinal and choroidal thickness were observed in the macular area of eyes diagnosed with RPC with macular involvement compared with the asymptomatic healthy fellow eyes. Further prospective studies are warranted in order to investigate the role of the choroid in cases of RPC.

## 1 Background

Relentless placoid chorioretinitis (RPC), also known as ampiginous choroiditis, was first described by Jones et al. as a particular type of posterior uveitis that shared clinical and angiographic characteristics with acute posterior multifocal placoid pigment epitheliopathy (APMPPE) and serpiginous choroiditis. However, it typically exhibits a prolonged clinical course, with recurrences of the inflammation after months to years following the onset [1]-[3]. The scattered distribution of the multiple, plaque-like, creamy coalescent lesions, affecting simultaneously the posterior pole and the midperipheral retina, usually leads to poor visual outcomes [1].

The etiopathogenesis of RPC remains unknown, but both fluorescein and indocyanine green angiographic findings evidence involvement of the choroid altogether with the typical retinal changes [1]. The analysis of the choroidal morphologic and morphometric features has significantly improved with the development of high-penetrance optical coherence tomography (OCT) devices [4],[5]. The last generation of these integrates the swept-source (SS) laser technology. The SS-OCT is characterized by a light source with a longer wavelength of 1,050 nm. This technical change allows deeper penetration through the ocular tissue, thus obtaining a three-dimensional (3D) high-contrast image of the retina and the choroid [6],[7]. This system has a scanning speed of 100,000 A-scans per second and a scan window depth of 2.6 mm with an axial resolution of 8 mm and a transverse resolution of 20 mm in tissue [8]. In the present study, we evaluated the choroidal thickness of the macular area in patients with unilateral RPC and macular involvement.

## 2 Methods

This observational retrospective study evaluated patients diagnosed with macular involvement due to unilateral RPC by a single physician (RGP) according to the previous reports on this disease [1]-[3]. The present study was conducted in accordance with the principles of the Declaration of Helsinki and in compliance with the La Fe University and Polytechnic Hospital review board and informed consent regulations.

**Figure 1:**
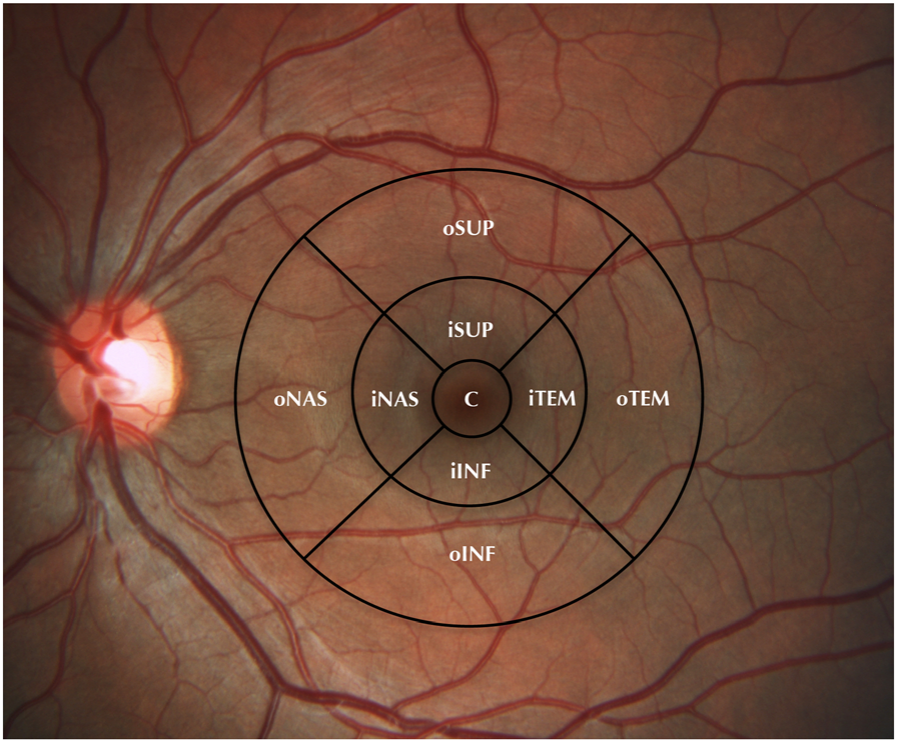
**Modified ETDRS grid (6 × 6 mm) overlapped to the fundus color photography.** This was done in order to analyze the retinal and choroidal thickness. The grid was subdivided in nine independent sectors: a central sector (C) within 500 μm from the fovea, four inner sectors within 500 to 1,500 μm from the fovea (iSUP, iTEM, iINF, iNAS), and four outer sectors within 1,500 to 3,000 μm from the fovea (oSUP, oTEM, oINF, oNAS).

We performed a comprehensive ophthalmic examination including best-corrected visual acuity (BCVA) using standard early treatment of diabetic retinopathy study (ETDRS) charts (decimal scale), axial length (AL) measurement (IOLMaster 500, Carl Zeiss Meditec, Inc., Dublin, CA, USA), slit-lamp examination, and color fundus and autofluorescence photographs (Visupac, Carl Zeiss Meditec Inc.). The macular region of all patients was scanned with SS-OCT (DRI OCT-1 Atlantis, Topcon, Tokyo, Japan) at a 1,050-nm wavelength with a 3D raster scan protocol. The SS-OCT 3D scan modality produces a retinal and choroidal thickness map of the macular area (12 × 9 mm) after an automated segmentation of the retina and the choroid. The retinal and choroidal thickness maps were overlap to the modified ETDRS grid (6 × 6 mm) to study the different mean values of each sector (Figure 1). The mean thickness of each sector was automatically measured within 500 μm from the center of the fovea in the central sector; 500 to 1,500 μm from the center of the fovea in four juxtafoveal or inner sectors (superior, inferior, nasal, and temporal), and 1,500 to 3,000 μm from the center of the fovea in four extrafoveal or outer sectors (superior, inferior, temporal, and nasal).

All the data were processed and a comparison between the data of the affected eye and the fellow healthy eye was performed. Statistical analysis was performed using Statistical Package for Social Sciences v.20.0 (IBM Corp., Armonk, NY, USA).

## 3 Results

**Figure 2:**
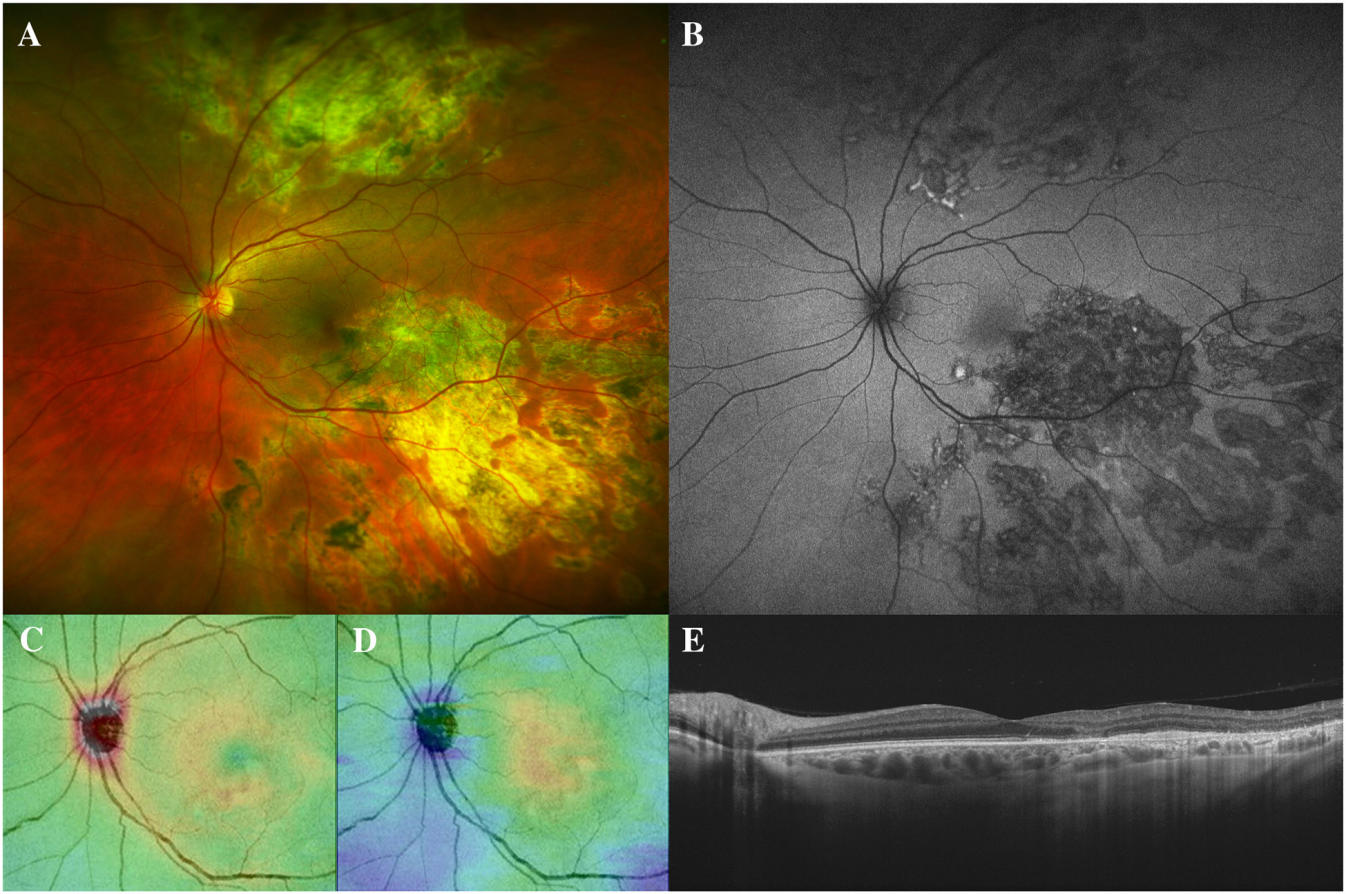
**Patient 1: a 62-year-old woman diagnosed with unilateral relentless placoid chorioretinitis affecting her left eye.** The color fundus and autofluorescence images **(A-B)** evidence multiple coalescent atrophic lesions affecting the posterior pole and the periphery of the retina. The retinal **(C)** and choroidal **(D)** thickness maps show normal measures. The swept-source horizontal optical coherence tomography scan centered in the fovea **(E)** demonstrates atrophic changes in the RPE in the temporal macular region associated with areas of disruption of the outer retina. The choroid shows no tomographic alteration in the macular area, whereas an indirect hyper-reflectivity is seen in the areas of RPE atrophy.

**Figure 3:**
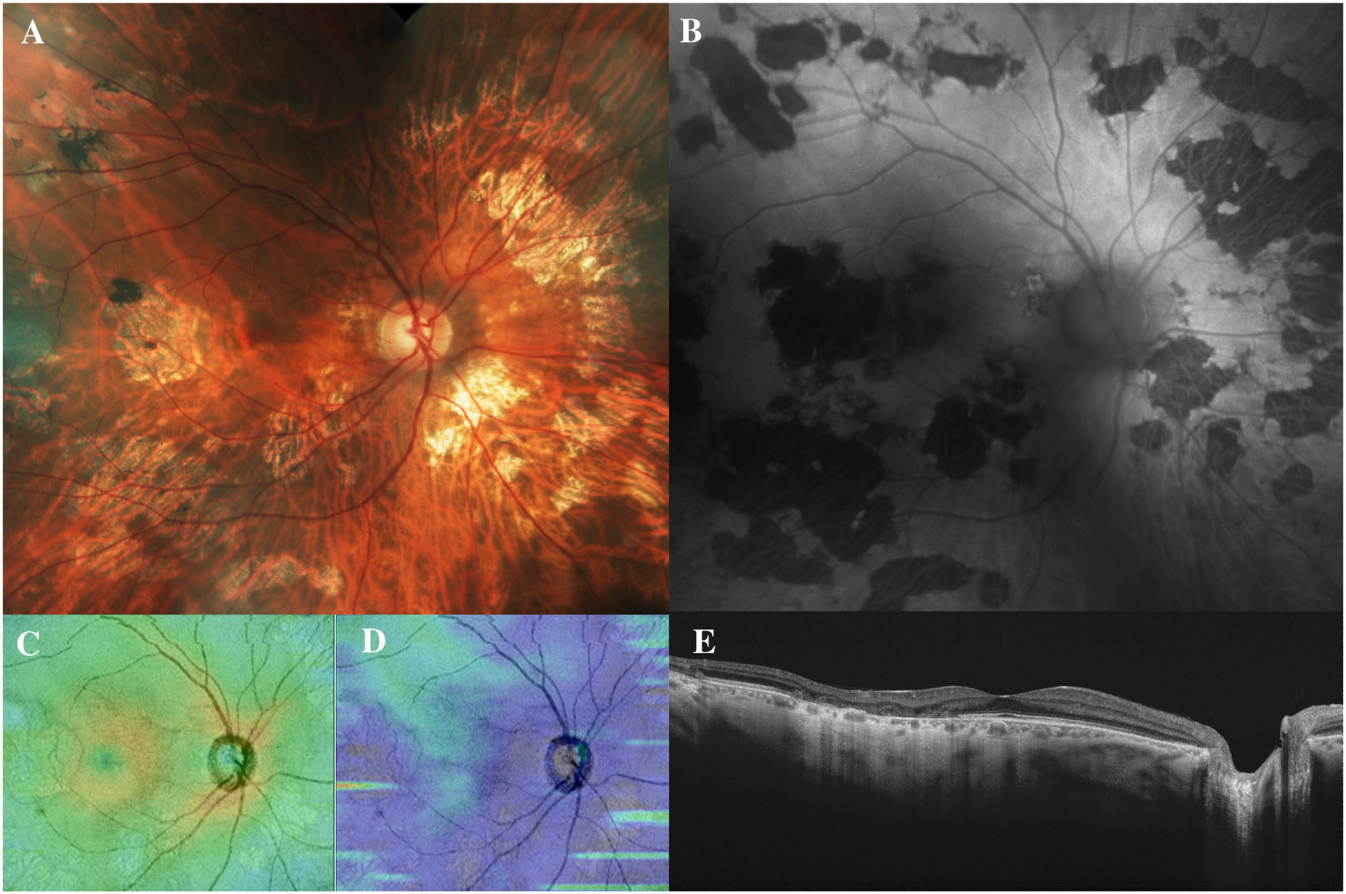
**Patient 2: a 50-year-old man diagnosed with unilateral relentless placoid chorioretinitis affecting his right eye.** The color fundus and autofluorescence images **(A-B)** demonstrate typical atrophic lesions involving the macula and also distributed widespread in the mid and far retinal periphery. The retinal **(C)** and choroidal **(D)** thickness maps evidence normal topographic measures. The swept-source horizontal optical coherence tomography scan centered in the fovea **(E)** shows a temporal window defect consistent with the atrophy of the RPE in the temporal macular region and a diffuse choroidal thinning.

**Figure 4:**
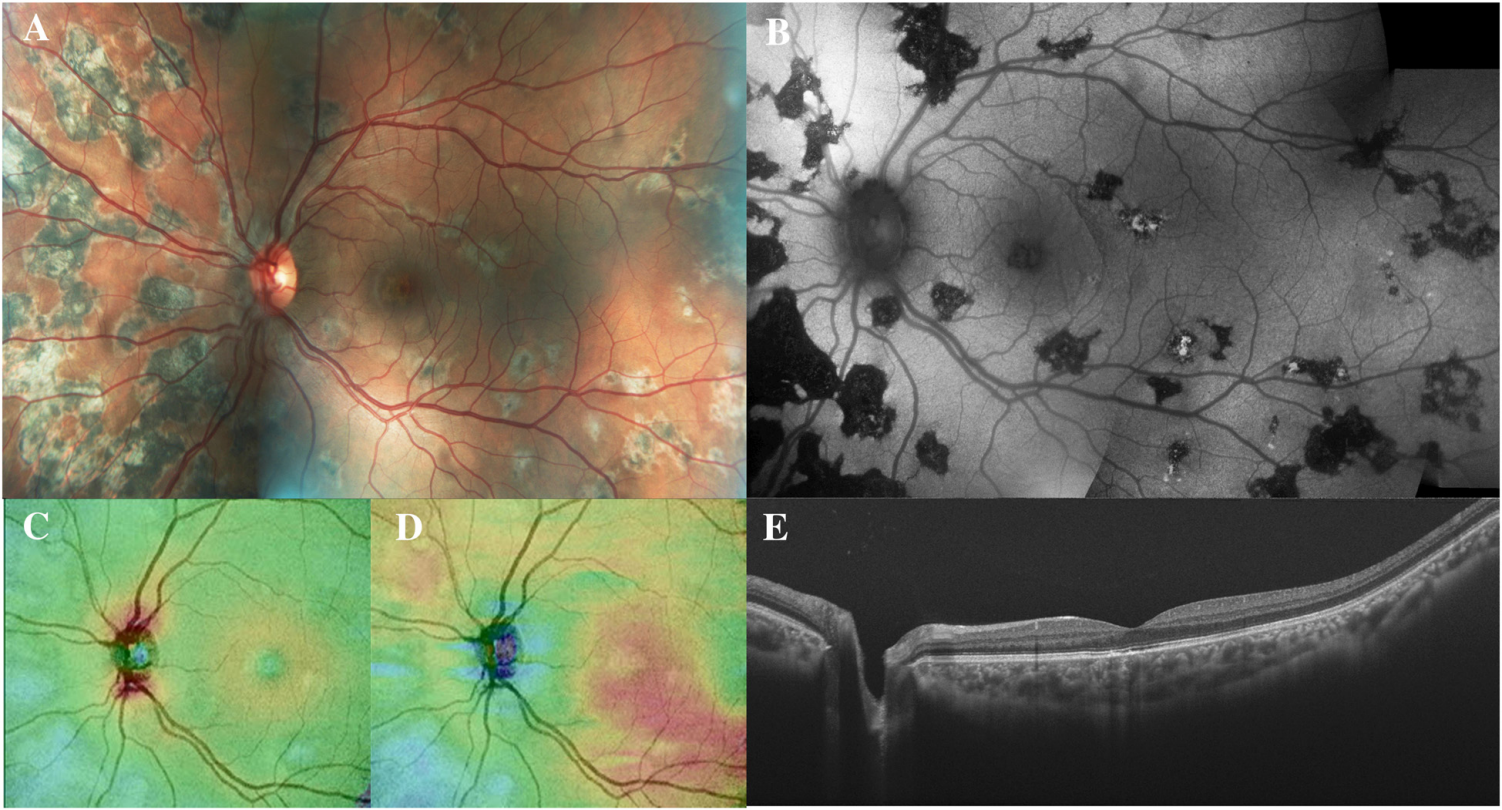
**Patient 3: a 17-year-old boy diagnosed with unilateral relentless placoid chorioretinitis affecting his left eye.** The color fundus and autofluorescence images **(A-B)** demonstrate multiple lesions widespread distributed throughout the retina, predominantly in the nasal region and involving the macula. The retinal **(C)** and choroidal **(D)** thickness maps evidence normal topographic measures. The swept-source horizontal optical coherence tomography scan centered in the fovea **(E)** shows minimal RPE changes with a normal choroidal tissue.

Three patients (two men aged 50 and 17 years and one woman aged 62 years) diagnosed with RPC and macular involvement were included (Figures 2, 3, and 4). The time from the onset of visual symptoms related to RPC was 56, 8, and 4 months for each case, respectively. Eyes with clinically evident RPC had a mean AL of 24.62 ± 0.11 mm, whereas in the clinically healthy fellow eyes, AL was 24.65 ± 0.03 mm (*p* = 0.70). The mean BCVA was 0.93 ± 0.16 (range 0.80 to 1.0) in the eyes with RPC, and 1.0 in all the fellow eyes (*p* = 0.70). The slit-lamp examination ruled out the presence of vitreous or anterior chamber inflammatory cells in all cases. All patients had been treated with oral steroids in a tapering regimen starting from a maximum dose of 90 mg per day (1 mg/kg/day). By the time of the SS-OCT examination, none of them was under steroid therapy (minimum 1 month after steroid cessation).

### 3.1 Quantitative analysis of the retina

The mean automatically measured retinal thickness of the macular area was 288.10 ± 10.22 μm in eyes with RPC and 300.30 ± 7.17 μm in the healthy fellow eyes; no statistically significant differences were evidenced (*p* = 0.20). The mean automatically measured retinal thickness in the central sector of the ETDRS grid (within 500 μm from the center of the fovea) was 257.33 ± 31.53 μm in eyes with RPC, and 269.33 ± 19.75 μm in the healthy fellow eyes; no statistically significant differences were observed (*p* = 0.99). The mean retinal thicknesses of the nasal, inferior, temporal, and superior sectors automatically measured in the juxtafoveal area (500 to 1,500 μm from the center of the fovea) and in the extrafoveal area (1,500 to 3,000 μm from the center of the fovea) are summarized in the Table 1.

**Table 1:** Mean retinal thickness values per sector

**Sector**	**Retina**		
	**Study eyes**	**Fellow eyes**	***p*** **value**
Central	257.33 ± 31.53	269.33 ± 19.76	0.827
Juxtafoveal nasal	310.00 ± 23.58	316.33 ± 17.95	0.658
Juxtafoveal inferior	283.67 ± 24.13	289.30 ± 4.62	0.825
Juxtafoveal temporal	310.67 ± 10.69	327.70 ± 9.50	0.127
Juxtafoveal superior	266.00 ± 8.88	271.70 ± 24.79	0.827
Extrafoveal nasal	308.00 ± 11.27	324.00 ± 5.00	0.127
Extrafoveal inferior	270.33 ± 15.04	289.99 ± 32.05	0.275
Extrafoveal temporal	316.33 ± 10.02	328.70 ± 6.66	0.127
Extrafoveal superior	270.33 ± 6.11	287.00 ± 18.52	0.127
Mean	288.07 ± 10.22	300.30 ± 7.17	0.127

### 3.2 Quantitative analysis of the choroid

The mean automatically measured choroidal thickness of the entire macular region was 248.60 ± 128.40 μm in eyes with RPC, and 255.10 ± 123.60 μm in the healthy fellow eyes; no statistically significant differences were observed (*p* = 0.99). The mean automatically measured choroidal thickness in the central sector of the ETDRS grid (within 500 μm from the center of the fovea) was 260.70 ± 140.60 μm in the study eyes, and 262.30 ± 123.10 μm in the healthy fellow eyes; no statistically significant differences were evidenced (*p* = 0.99). The mean automatically measured choroidal thickness in the juxtafoveal sectors (500 to 1,500 μm from the center of the fovea) and extrafoveal sectors (1,500 to 3,000 μm from the center of the fovea) showed no significant differences between the affected eyes and the healthy fellow eyes as reported in the Table 2.

**Table 2:** Mean choroidal thickness values per sector

**Sector**	**Choroid**		
	**Study eyes**	**Fellow eyes**	***p*** **value**
Central	260.67 ± 140.61	262.33 ± 123.09	0.827
Juxtafoveal nasal	260.68 ± 137.70	247.33 ± 142.42	0.658
Juxtafoveal inferior	260.69 ± 76.36	198.00 ± 122.39	0.827
Juxtafoveal temporal	260.70 ± 155.02	256.33 ± 134.93	0.827
Juxtafoveal superior	260.71 ± 163.02	238.67 ± 135.55	0.827
Extrafoveal nasal	260.72 ± 146.32	271.67 ± 117.46	0.827
Extrafoveal inferior	260.73 ± 132.20	250.00 ± 113.22	0.513
Extrafoveal temporal	260.74 ± 124.94	287.67 ± 125.64	0.827
Extrafoveal superior	260.75 ± 100.37	283.67 ± 129.08	0.827
Mean	260.76 ± 128.44	255.07 ± 123.62	0.827

## 4 Discussion

The differential diagnosis of RPC includes mainly APMPPE and serpiginous choroiditis. These diseases share similar clinical and angiographic characteristics, with creamy lesions that exhibit early hypofluorescence in the fluorescein angiography with later staining [1]. The different clinical course and the atypical distribution of the lesions in patients with RPC may assist its diagnosis, although some cases may need a long-term follow-up to be able to differentiate them.

The angiographic characteristics in patients with acute RPC reveal a presumed choroidal involvement, which represent a common finding in cases of posterior uveitis. Changes in the choroidal thickness have been reported in many inflammatory diseases as Vogt-Koyanagi-Harada disease [8], Behçet uveitis [9] or posterior scleritis [10]. To the best of our knowledge, there are no reports in the literature regarding the influence of RPC, APMPPE, or serpiginous choroiditis in the choroidal tissue.

## 5 Conclusions

We studied the retinal and choroidal thickness of the macula in cases of unilateral RPC with macular involvement in a quiescent stage. Our results did not evidence any difference in the retinal or choroidal thickness in the macular area when comparing the affected eye with the fellow healthy eye. We hypothesized that this characteristic may help in the differential diagnosis with serpiginous choroiditis, since cases of serpiginous choroiditis may lead to severe atrophic legacy not only in the retina but also in the choroid. This relative sparing of the choroid in RPC may be related to the degree of the inflammatory reaction on the tissue.

The limitations of our study include the short number of patients and follow-up. There is also a lack of reports regarding the choroidal thickness in cases of posterior uveitis. Further studies are warranted in order to confirm the sparing of the choroidal thickness in cases of RPC. These data might be useful in the differentiation of plaque-like white dot syndromes as RPC, APMPPE, and serpiginous choroiditis.

## Abbreviations

3D: Three dimension

AL: Axial length

APMPPE: Acute posterior multifocal placoid pigment epitheliopathy

ETDRS: Standard early treatment of diabetic retinopathy study

OCT: Optical coherence tomography

RPC: Relentless placoid chorioretinitis

SS: Swept-source

## Competing interests

RDM received research grants from Alcon, Allergan, Bayer, Heidelberg Engineering, Novartis, and Thea. RGP is a consultant to Alcon, Bayer, and Novartis and received research grants from Alcon, Allergan, Bayer, Heidelberg Engineering, Novartis, Sensimed, and Thea. The other authors declare that they have no competing interests.

## Authors' contributions

All authors have made substantial contributions to conception and design of the study or acquisition, analysis, and interpretation of data; have been involved in drafting the manuscript or revising it critically for important intellectual content; have given final approval of the version to be published; and agreed to be accountable for all aspects of the work in ensuring that questions related to the accuracy or integrity of any part of the work are appropriately investigated and resolved. All authors read and approved the final manuscript.

## Anknowledgements

We thank Nestor García Marín for his valuable help in the examination of patients and Dr. M Dolores Pinazo Durán for the evaluation and critical analysis of the study design.
